# Vectorization by nanoparticles decreases the overall toxicity of airborne pollutants

**DOI:** 10.1371/journal.pone.0183243

**Published:** 2017-08-15

**Authors:** Rodolphe Carpentier, Anne Platel, Helena Maiz-Gregores, Fabrice Nesslany, Didier Betbeder

**Affiliations:** 1 Inserm, LIRIC - UMR 995, Lille, France; 2 Univ Lille, LIRIC - UMR 995, Lille, France; 3 CHRU de Lille, LIRIC - UMR 995, Lille, France; 4 Institut Pasteur de Lille, Laboratoire de Toxicologie Génétique, Lille, France; 5 Univ Lille, EA4483, Lille, France; 6 Université d’Artois, Lens, France; VIT University, INDIA

## Abstract

Atmospheric pollution is mainly composed of volatile pollutants and particulate matter that strongly interact. However, their specific roles in the induction of cellular toxicity, in particular the impact of the vectorization of atmospheric pollutants by ultrafine particles, remains to be fully elucidated. For this purpose, non-toxic poly-lactic co-glycolic acid (PLGA) nanoparticles were synthesized and three pollutants (benzo(a)pyrene, naphthalene and di-ethyl-hexyl-phthalate) were adsorbed on the surface of the nanoparticles in order to evaluate the toxicity (cytotoxicity, genotoxicity and ROS induction) of these complexes to a human airway epithelial cell line. The adsorption of the pollutants onto the nanoparticles was confirmed by HPLC analysis. Interestingly, the cytotoxicity assays (MTT, LDH and CellTox Green) clearly demonstrated that the vectorization by nanoparticles decreases the toxicity of the adsorbed pollutants. Genotoxicity was assessed by the micronucleus test and the comet assay and showed no increase in primary DNA damage or in chromosomal aberrations of nanoparticle vectorized pollutants. Neither cytotoxicity nor genotoxicity was correlated with ROS induction. To conclude, our results indicate that the vectorization of pollutants by nanoparticles does not potentiate the toxicity of the pollutants studied and that, on the contrary, adsorption onto nanoparticles could protect cells against pollutants’ toxicity.

## Introduction

Air quality remains a major issue but, while evidence continues to accumulate as to the adverse health effects of air pollution, the main mechanisms driving these effects remain poorly understood. Urban and industrial air pollution are composed of gases (more or less volatile) and particulate matter (PM). Atmospheric particles can be categorized according to their aerodynamic diameter: PM10 for particles equal or smaller than 10μm, PM2.5 for those equal or smaller than 2.5μm, and ultrafine particles (UFP) for those smaller than 100nm (or nanoparticles (NP) since the growth of nanotechnology).

Atmospheric particles have diverse origins. Naturally produced particles include dusts from erosion, volcanic eruptions or forest fires and represent 90% of the total particulates in the ambient atmosphere [1]. Anthropic particles are generated by cars, during charcoal burning, by industry or the cooking of food, etc and some are also intentionally produced for cosmetics, pharmacology or medicine applications. In urban areas, diesel and automobile exhausts are the primary source of atmospheric particles [2], and most particles released from vehicles are in the size range of 20–130 nm [3, 4].

While UFP represent less than 8% of the total mass of atmospheric particles, they dominate the total number of particles [5, 6]. The small size of UFP increases their specific surface area compared to fine or coarse particles and thus heightens their potential reactivity with pollutants in the gas phase. Consequently, UFP may behave as vectors for pollutants through adsorption of volatile and semi-volatile organic compounds [7–10]. The complex mixture of gaseous pollutants and particulates in ambient air lead to adverse health effects, though the precise role of each component in human toxicity remains to be elucidated. Some studies on airborne pollutants incriminate the gas phase [11–13], while others designate the particulate matter [14]. Moreover, synergies between gaseous phase pollutants and particulate matter are also described and provoke questions as to the role of interactions between these components in toxicity [15–17]. Indeed, interactions between atmospheric particles and gases rely on the interplay between particle parameters (size, shape, surface charge, composition), gas properties (volatility, composition) and environment (temperature, humidity, sun light), making the evaluation of the toxicity of air pollution extremely challenging.

One of the main exposure routes to air pollutants is inhalation. Owing to their small size, UFP can penetrate deep into the lungs and are thought to be responsible for the observed alveolar toxicity and mortality [5, 18–26], supporting by the deposition mechanism of the inhaled UFP. However, considering the available surface of the conducting airways, the deposition efficiency of UFP into bronchia is also very high [9].

UFP may act as vectors for gaseous pollutants and their small size results in their accumulation in cells via, on the one hand escaping mucociliary clearance, and on the other through their endocytosis by respiratory epithelial cells and alveolar macrophages [9, 22, 27, 28]. However, the real contribution of UFP to the vectorization of atmospheric gaseous pollutants into cells and the resulting toxicity thus provoked, has not been fully examined and is confounded by the fact that UFP have an intrinsic toxicity. Among the atmospheric gaseous pollutants, several groups of partially volatile pollutants exist such as dioxins, ozone, nitrogen oxides and sulfur oxides, phthalates, or polycyclic aromatic hydrocarbons (PAH); these latter are one of the most-studied contributors to the toxicity of atmospheric pollution. Atmospheric PAH originate from incomplete combustion of organic material such as wood, coal, oil and refinery products, tobacco, etc [29]. Phthalates are used as plasticizers and can leach from plastics; they are found in building materials and furniture, including upholstery, mattresses, wall coverings, floor tiles and vinyl flooring, but also in food packaging and cosmetics.

In an attempt to decipher the role of UFP-driven vectorization on the toxicity of gaseous pollutants, we produced non-toxic nanoparticles based on the well-described FDA approved copolymer PLGA (Poly Lactic co-Glycolic Acid) [30], and analyzed the cyto- and geno-toxicities of three free or nanoparticle-adsorbed pollutants on a selected airway epithelial model, the NCI-H292 cell line.

We chose to include two well-known carcinogenic PAH, Naphthalene (NA) and Benzo(a)pyrene (B(a)P) [31, 32], and one endocrine disruptor of the phthalate family, Di-Ethyl-Hexyl-Phthalate (DEHP), hereafter referred to collectively as “the pollutant(s)”. NA is one of the most abundant PAHs in ambient air, present almost exclusively in the vapor phase, and is classified as a possible carcinogen (IARC group 2B, C2 by CLP). In contrast, B(a)P exists primarily in the particle-bound state in the environment and is a known carcinogen (IARC group 1, C1B M1B R1B by CLP) with a relatively well-understood mechanisms of toxicity [33]. Finally, DEHP is highly present in indoor air and is a known endocrine disruptor and possible carcinogen (IARC group 2B by, R1B by CLP) [34]. The *in vitro* toxicity of these three pollutants, either free or adsorbed onto non-toxic nanoparticles, was determined using a variety of biological endpoints including ROS measurements, cytotoxicity and genotoxicity assays.

## Methods

### Reagents and chemicals

RPMI 1640, horse serum, fetal calf serum (FCS), non-essential amino acids and phosphate buffered saline (PBS) were purchased from GIBCO Invitrogen (Cergy-Pontoise, France). PolyLactic coGlycolic Acid copolymer (PLGA, Resomer^®^ RG503H) was from Evonik (Essen, Deutschland). Benzo(a)pyrene, naphthalene, di-ethyl-hexyl phthalate, referred as ‘pollutant’ and H2-DCFDA, pyocyanine, giemsa reagent, penicillin, streptomycin, amphotericin B, cyclophosphamide (CPA, CAS No. 6055-19-2), mitomycin C (MitoC, CAS No. 50-07-7), methyl methane sulfonate (MMS, CAS No. 66-27-3), Triton X-100, EDTA, HEPES, trizma base, propidium iodide, KCl, NaCl, sodium bicarbonate, sodium pyruvate, pluronic F68 solution, Trypan Blue, bovine serum albumin (BSA) were obtained from Sigma-Aldrich (Saint-Quentin Fallavier, France). Normal melting point agarose and low melting point agarose were purchased from Bio-rad (Marnes-la-Coquette, France). Acetic acid was purchased from VWR (Fontenay sous-bois, France). Dimethyl sulfoxide (DMSO) from Acros Organics (Noisy le Grand, France). Trypsin was obtained from Biochrom AG (Berlin, Germany), while NaOH, L-glutamine and absolute ethanol were from Merck (Darmstadt, Germany). The repair endonuclease hOgg1 was purchased from New England Bio-Labs via Ozyme (Saint Quentin en Yvelines, France).

### Formulation and analysis

Nanoparticles of poly lactic-co-glycolic acid (PLGA(-)) were synthetized by nanoprecipitation without surfactant or detergent as previously described [30, 35]. The PLGA was dissolved at 10mg.ml^-1^ in a mixture of acetone and ethanol (85/15: v/v) and injected in ultrapure water under stirring at 150rpm at 21°c. After 30 minutes, residual organic solvents were eliminated by vacuum evaporation at 27°c to produce PLGA(-) nanoparticles in ultrapure water at 1mg.ml^-1^.

Adsorption of the pollutants was performed by a single-step mixing. They were dissolved in acetone and injected to the solution of PLGA(-).

The average hydrodynamic diameter of PLGA(-) and pollutant formulations were measured in 15 mM NaCl by dynamic light scattering using a Zetasizer NanoZS instrument. The polydispersity index (PDI) represent the homogeneity of the size dispersion of a nanoparticle population and nanoparticles are considered monodisperse for values below 0.3.

The zeta potential of PLGA(-) and formulations were determined by electrophoretic mobility in water on a Zetasizer NanoZS instrument (Malvern Instruments, Orsay, France).

### Association efficiency of pollutants on PLGA nanoparticles

Free *versus* associated pollutants were separated by centrifugation of the formulations on a 10kDa NanoSep^®^ (Pall, France) at 5.000g for 5 min. The amount of pollutants in the filtrates were quantified by high-performance liquid chromatography (HPLC) using calibration curves of free pollutants. The percentage of association efficiency (AE) was calculated as follows:

$$\%AE=\;\;\left[\frac{theoretical\;pollutant\;amount-calculated\;pollutant\;in\;the\;filtrate}{theoretical\;pollutant\;amount}\right]\;\times \;100$$

The chromatographic analyses were performed on an Ultimate 3000 Autosampler (ThermoScientific, France) and chromatographic data were collected and processed with the Chromeleon software.

The analytical column (Accucore aQ C18, 150x3 mm, 2.6μm particle size) was purchased from ThermoScientific, France. The flow rate was 0.5 mL.min-1 and the injection volume was 100μL. For naphthalene the mobile phase consisted of methanol/water (70:30 v/v) with a detection at 219nm and a retention time around 5 min. For benzo(a)pyrene, the mobile phase consisted of methanol/water (90:10,v/v) with a detection at 254nm and a retention time around 8 min. For DEHP, the mobile phase was a gradient of water/acetonitrile (15:85 v/v) progressively shifting to 100% acetonitrile (from 3 to 6.5 min) The detection was at 225nm with a retention time of around 9 min.

### Preparation of S9 mix

Extracellular metabolic activation of pollutants was performed by using the S9 fraction from male Sprague Dawley rat livers induced by beta-naphthoflavone and phenobarbital (Biopredic, France). The S9 mix consisted of 200mM of phosphate buffer pH 7.4 (NaH2PO4 0.2M and Na2HPO4 0.2M), 40mM of beta-nicotinamide-adenine-dinucleotide-phosphate hydrate, 5mM of glucose-6-phosphate, 33.3mM of MgCl2, 8mM of KCl (Sigma Aldrich, France) and 5% (v:v) of S9 fraction.

### Cell culture and treatment schedule for cytotoxicity

The human muco-epidermoid bronchiolar carcinoma cell line NCI-H292 was generously donated by Dr J.M. Lo-Guidice (EA4483, Université de Lille 2, Lille, France). Cells were maintained in RPMI1640 medium supplemented with 10% fetal bovine serum at 37°C under 5% CO_2_ atmosphere.

Cells were seeded in 96-well plate at a concentration of 2.10^4^ cells/well. After 72h, cells were treated with formulations for 15 hours (S9- 15h) or for 3 hours, followed by 15 hours of recovery (3h/15h) with S9 mix metabolic activation (S9+ 3h/15h), or not (S9- 3h/15h).

### Cytotoxicity

Cytotoxicity tests were multiplexed depending on the treatment. For the long treatment (S9- 15h), cell mortality was first performed by DNA dye exclusion assay then viability was determined by MTT-based assay. For the short treatment (S9+ 3h/15h and S9- 3h/15h), mortality and viability were successively assessed by LDH content determination on cell culture supernatants and MTT-based assay on cell cultures.

The DNA dye exclusion mortality test was done with the CellTox Green^®^ cytotoxicity assay (Promega, France) following manufacturer’s instructions. Briefly, 0.15% (v/v) of CellTox Green dye^®^ was incubated with cells for 30 minutes at 37°C. The dye fixes released DNA from dead cells or enters dead cells to bind DNA. Dye fluorescence was quantitative for the number of dead cells and was read on a Fluoroskan Ascent^®^ (ThermoScientific, France) with excitation/emission wavelengths of 488nm/527nm, respectively. Positive control (100% of mortality) was by a treatment with 10% Triton X100 in PBS while the negative control (0% of mortality) was determined on untreated cells.

The LDH content was determined with the CytoTox 96^®^ non-radioactive cytotoxicity assay kit (Promega, France), a LDH-induced tetrazolium-to-formazan conversion, and was used according to the manufacturer’s instructions. Supernatants were clarified by centrifugation (100g, 5min, room temperature). The reaction was performed for 30 min at 37°C then stopped and absorbance were read at 490nm on a MultiSkan Go^®^ spectrophotometer. Positive control (100% of mortality) was established by a treatment with 10% Triton X100 in PBS while the negative control (0% of mortality) was performed on untreated cells.

The MTT-based viability test was adapted from the CellTiter 96^®^ non-radioactive cell proliferation assay, according to the manufacturer’s instructions. Cells were washed with PBS and 15% (v/v) of dye solution were added for 3 hours at 37°C then stopped. After 1 hour, absorbance was read at 590nm on a MultiSkan Go^®^ spectrophotometer (ThermoScientific, France). Negative control (100% viability) was untreated cells while 4% (v/v) paraformaldehyde (20min at 37°C) was used as positive control (0% viability).

### Reactive oxygen species (ROS) measurement

Intracellular ROS induction was measured by H2-DCF-DA probe. Cells were seeded in 96-well plate at a concentration of 2.10^4^ cells/well for 72 hours. After 2 PBS washes, 10μM H2-DCF-DA in PBS were added to the cells for 60 minutes at 37°C. Cells were washed again and nanoparticles, pollutants or formulations were added and the ROS-induced oxidation of the probe was measured following its 488nm/527nm fluorescence using a FluoroSkan Ascent^®^ (ThermoScientific, France). Pyocyanine was used as a positive control.

### Assessment of primary DNA damage by the comet assay

Cells were seeded at a density of 3x10^5^ cells/well (3 ml culture medium/well) in 6-well cell culture plates (9.6 cm^2^, Falcon^™^, Becton Dickinson Biosciences, France) and incubated for 48 h prior to treatment in order to allow exponential cell growth and maximum cell attachment. Cells were treated for 3 h without recovery time (3h/+0h) with nanoparticles, pollutants or formulations, with a metabolic activation step (S9+) for B(a)P and NA, then collected by trypsinisation. MMS (15 μg.ml^-1^) was used as a positive control for experiments without metabolic activation and B(a)P (100μM) for S9+ conditions.

The cytotoxicity was assessed using the Trypan Blue dye exclusion assay. Cell suspensions were gently mixed with Trypan Blue solution (0.08%, v/v) and scored using a Malassez hemacytometer. A viability percent higher than 70% were submitted to the comet assay.

The comet assay was performed under alkaline conditions (pH>13) as described by Singh *et al*. [36]. Minor modifications were done to specifically detect oxidative DNA damage, based on Collins’ and Smith’s procedures [37, 38]. At the end of the treatment period, 8 to 10.10^4^ viable cells from each well were mixed with 75 μl of 0.5% wt/v low-melting point agarose kept at 37°C, before rapidly transferring onto pre-coated slides (two layers of normal agarose (1.5% and 0.8%, wt/v). Each slide was duplicated for the treatment either with the enzyme buffer or with hOgg1 enzyme. All steps were sheltered from daylight to prevent the occurrence of additional DNA damage. Slides were immersed for at least 1 h at 4°C in a cold lysing solution (2.5 M NaCl, 100 mM EDTA, 10 mM Trizma Base, pH 10, supplemented with 1% Triton X-100, v/v and 10% DMSO, v/v). After lysis, slides were washed and equilibrated for 2 x 5 min in enzyme buffer (40 mM HEPES, 100 mM KCl, 0.5 mM EDTA, 0.2 mg.ml^-1^ BSA, pH 8).

Each slide was then treated with 75 μL of enzyme buffer alone, or hOgg1 solution (0.12 units per gel) before incubation in a humidified box at 37°C for 20 min or 10 min, respectively. Slides were quickly rinsed with cold PBS then placed in an electrophoresis tank previously filled with fresh electrophoresis solution (1 mM EDTA and 300 mM NaOH, pH>13) and left in the solution for 20 min to allow DNA unwinding and expression of single-strand breaks and alkali-labile sites. Next, electrophoresis was conducted at +4°C for 20 min, using an electric current of 0.7 V/cm (25 V/300 mA). After electrophoresis, slides were neutralized for 2 x 5 min in a neutralization solution (0.4 M Tris, pH 7.5), and gels were dehydrated by immersion in absolute ethanol for 5 min. Finally, slides were air-dried and stained by addition of 25 μl of propidium iodide (20 μg.ml^-1^ in distilled water) and a coverslip. Slides were then examined at 250x magnification using a fluorescence microscope (Leica Microscopy and Scientific Instruments Group, Heerbrugg, Switzerland) equipped with an excitation filter of 515–560 nm and a 590 nm barrier filter, connected through a gated CCD camera to Comet Image Analysis System software (version 4, Perceptive Instruments Ltd., Haverhill, United Kingdom). Four hundred randomly selected cells per condition (100 cells from each of the 2 duplicate slides in the 2 cultures) were scored. Tail intensity, defined as the percentage of DNA that had migrated from the head of the comet into the tail, was used as the measure of DNA damage. The non-parametric Mann-Whitney U-test was used to evaluate the statistical difference between groups since the tail intensity measurement in the comet assay did not follow a Gaussian distribution [39]. The Mann-Whitney U-test was also used, for each dose, for the comparison between free and nanoparticle-adsorbed pollutants. Differences with a *p*<0.05 were considered statistically significant. All statistical analyses were performed with StatView^®^ Software (version 5.0, SAS Institute Inc., SAS Campus Drive, Cary, North Carolina 27513, USA).

### Assessment of chromosomal aberrations by the micronucleus test

The micronucleus test was performed following the OECD test guideline 487 [40]. Cells were treated for 3 h with nanoparticles, pollutants or formulations, with a metabolic activation step (S9+) for the B(a)P and the NA then washed with PBS and incubated at 37°C for an additional 48 h recovery period (3h/+48h). Cells were then collected by trypsinisation. Positive controls were MitoC (0.25 μg.ml^-1^) without metabolic activation or CPA (10 μg.ml^-1^) with S9 mix pre-treatment.

After the recovery period, cells were collected and treated for 7.5 min with a hypotonic solution (RPMI medium diluted 1:1 v/v in distilled water, 5 ml/tube). A pre-fixation step was performed by adding 500 μl/tube of cold Carnoy mixture (ethanol/acetic acid, 3:1 v/v). Cells were then centrifuged at 1000 rpm for 5 min and pelleted cells were fixed for at least 10 min at room temperature with 5 ml/tube of cold Carnoy mixture. After centrifugation, cells were spread on glass slides, air-dried at room temperature (at least over-night), stained for 10 min with 4% Giemsa water solution, rinsed with distilled water and finally dried at room temperature. Micronuclei (MN), identified according to recommended criteria [41–43], were scored in 1000 intact mononucleated cells per slide (2000 mononucleated cells/concentration) at 500x magnification. The statistical significance of difference between each concentration *vs* the negative control was determined using the CHI-squared test. The CHI-squared test was also used, for each dose, for the comparison between free and nanoparticle-adsorbed pollutants. Differences with a *p*<0.05 were considered statistically significant.

## Results

### Selection of the cellular model

The *in vitro* airway epithelial model was first selected based on the known toxicity of B(a)P. It was tested on three different airway epithelial cells, namely NCI-H292 (H292), A549 and 16HBE14o-. Cell viability was not affected by 1μM of B(a)P treatment in 16HBE14o- precluding this model in this study. A549 showed a reduced viability (of about 35%) while H292 presented a clear decrease (45%) (data not shown). We thus selected H292 cell line as a good candidate to test the modulation of toxicity by free or nanoparticle-adsorbed pollutants.

### Determination of the LC50 of the pollutants

The LC50 of each pollutant was evaluated by the MTT test on the H292 cells. NA is a common polycyclic aromatic hydrocarbon (PAH) possibly carcinogenic for Humans (IARC class 2B). The LC50 was evaluated at 500μM. B(a)P is a carcinogenic PAH for Humans (IARC class 1) and the LC50 was 50μM. Finally, the LC50 of the endocrine disruptor Di-Ethyl-Hexyl-Phthalate (DEHP), a possible carcinogen for Humans (IARC class 2B), was determined at 150μM (data not shown). These doses were retained for all the following experiments.

### Preparation of PLGA nanoparticles and characterization of pollutant adsorption

Anionic nanoparticles of poly lactic-co-glycolic acid (PLGA(-)) were synthetized by nanoprecipitation without the use of any surfactant or detergent [35]. This method produced PLGA(-) with a mean size of 60nm (59.50nm +/- 25.24) and a negative zeta potential (-28mV, Table 1).

**Table 1:** Zeta potential, size and polydispersity index (PDI) measurements.

	Zeta potential (mV)	Size (nm, +/-SD)	PDI
**PLGA(-)**	-28.0	59.50 +/-25.24	0.180
**PLGA(-) NA**	-45.8	48.06 +/-23.25	0.234
**PLGA(-) B(a)P**	-33.6	64.65 +/-30.80	0.227
**PLGA(-) DEHP**	-33.3	58.51 +/-23.69	0.164

Zeta potential, size and polydispersity index (PDI) of PLGA nanoparticles (PLGA(-)) alone, and associated with naphthalene (NA), benzo(a)pyrene (B(a)P) or di-ethyl-hexyl phthalate (DEHP) were measured by DLS (Zetasizer NanoZS, Malvern Instrument). PDI represents the size dispersity of the nanoparticule’s population and values below 0.3 are considered as monodisperse.

Pollutant-adsorbed PLGA nanoparticles were produced by simply mixing the pollutants with the nanoparticles in water. The adsorption took place rapidly, considering the hydrophobic surface properties of the nanoparticles.

The adsorption of NA onto PLGA(-) (PLGA(-) NA) decreased the zeta potential to -45mV without significant modification of the size of the PLGA(-) (48.06nm+/-23.25) (Table 1). This corresponded to a nanoparticle loading of 25.64% (w:w) and the resulting association efficiency was 99.94% as determined by HPLC (Table 2).

**Table 2:** Loading and association efficiency percentage of pollutants adsorbed on PLGA(-) nanoparticles.

	Loading	Association efficiency
**PLGA(-) NA**	25.64%	99.94%
**PLGA(-) B(a)P**	5.04%	100%
**PLGA(-) DEHP**	23.44%	85.40%

The loading of the three pollutants with PLGA(-) nanoparticles was expressed as a weight/weight percentage. The association efficiency (a.e.) of the three pollutants with the PLGA(-) nanoparticles was determined by HPLC and calculated as described in the Methods section.

Regarding B(a)P, the size of PLGA(-) was equivalent to PLGA(-) B(a)P (64.65nm +/-30.80) while the zeta potential appeared more negative (-33.6mV) (Table 1). The association efficiency was 100% as determined by HPLC for a loading of 5.04% (w:w) (Table 2).

Finally the size and the zeta potential of PLGA(-) DEHP were similar to PLGA(-) (58.51nm +/- 23.69; -33.3mV) (Table 1). This corresponded to a nanoparticle loading of 23.44% (w:w) with a loading efficiency of 85.4% (Table 2).

Together, these results show that the PLGA(-) and the nanoparticles with pollutant-adsorbed to their surfaces shared common characteristics in terms of size and surface charge, and were suitable for the toxicity studies.

### Cytotoxicity assays of PLGA(-)

**Fig 1:**
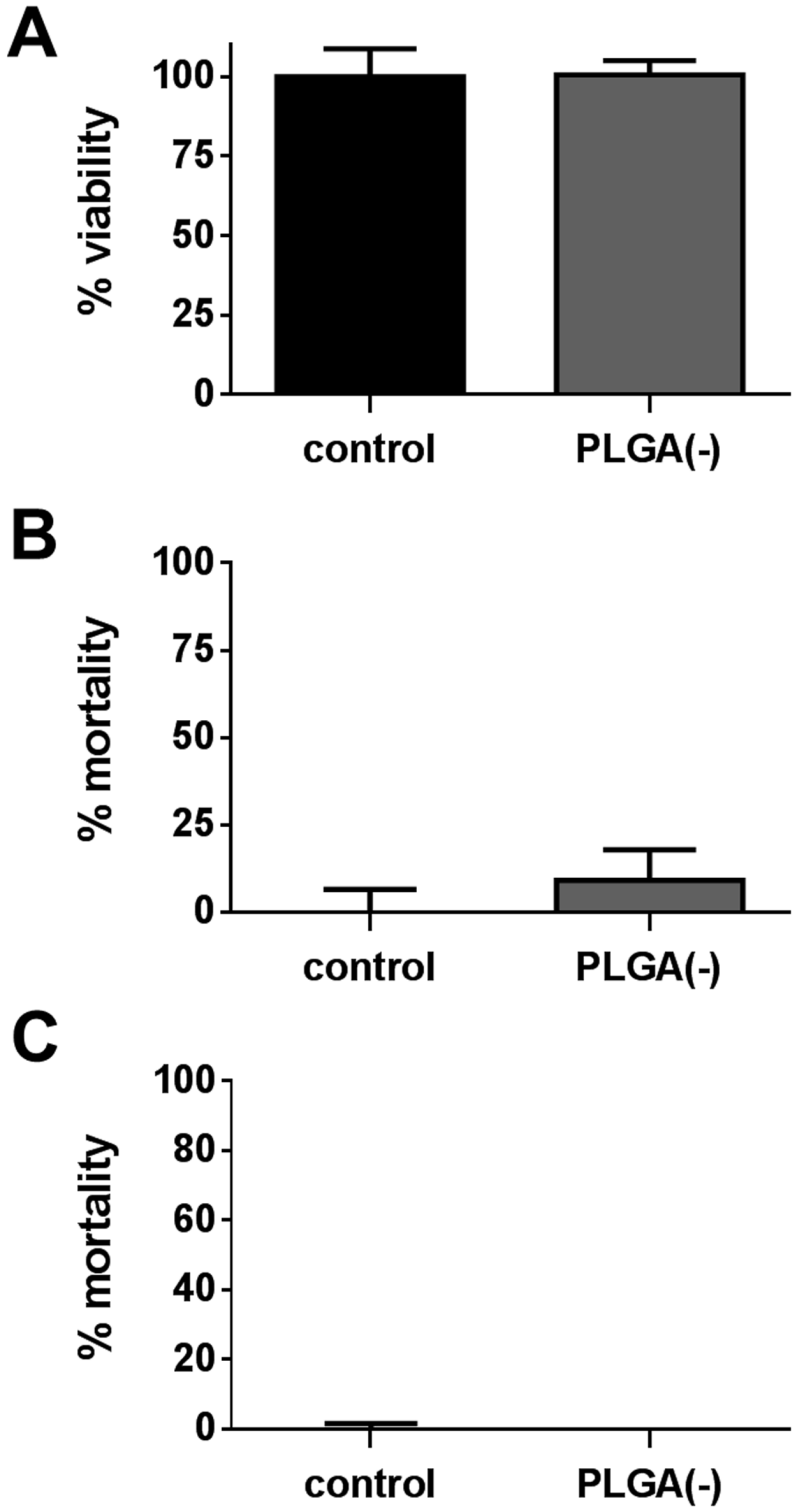
Evaluation of the cytotoxicity of PLGA(-) nanoparticles. H292 cells were treated for 15h with 250μg.ml^-1^ of PLGA(-) nanoparticles and cytotoxicity was assessed by MTT assay (A), LDH assay (B) and CellTox green assay^®^ (C) and are respectively presented as percentage of viability (A) and mortality (B and C).

Three tests were performed to determine the cytotoxicity of PLGA carriers on their own. The MTT assay depicts the mitochondrial activity and is considered as a viability test; the LDH assay measures the activity of lactate dehydrogenase (LDH) released from damaged cells; CellTox Green^®^ dye is excluded from viable cells and preferentially stains the DNA from dead cells. These last two assays are sensitive to plasma membrane integrity and are considered as mortality tests. However, the CellTox Green^®^ assay is more sensitive and suitable for long treatment (over 8h), unlike the LDH assay. Treatment of H292 cells with PLGA(-) at a final concentration of 250μg.ml^-1^ did not show any toxicity as assessed by the three cytotoxicity tests (Fig 1), making this carrier safe and acceptable for the following toxicity studies of the vectorized pollutants.

### Vectorization effect on naphthalene toxicity

#### Cytotoxicity study and ROS induction

NA can be metabolized by the P450 cytochrome family members and metabolites are known to exhibit toxicity. The activation of NA was realized *in vitro* by an incubation with the S9 fraction of rat liver homogenates (S9 mix). Free or PLGA(-)-associated NA (PLGA(-) NA) were loaded to H292 cells according to three protocols: a 3-hour exposure followed by a 15-hour period of recovery (S9- 3h/15h); a 3-hour exposure with S9 mix-activated pollutants followed by a 15-hour period of recovery (S9+ 3h/15h); or a continuous exposure of 15h (S9- 15h).

**Fig 2:**
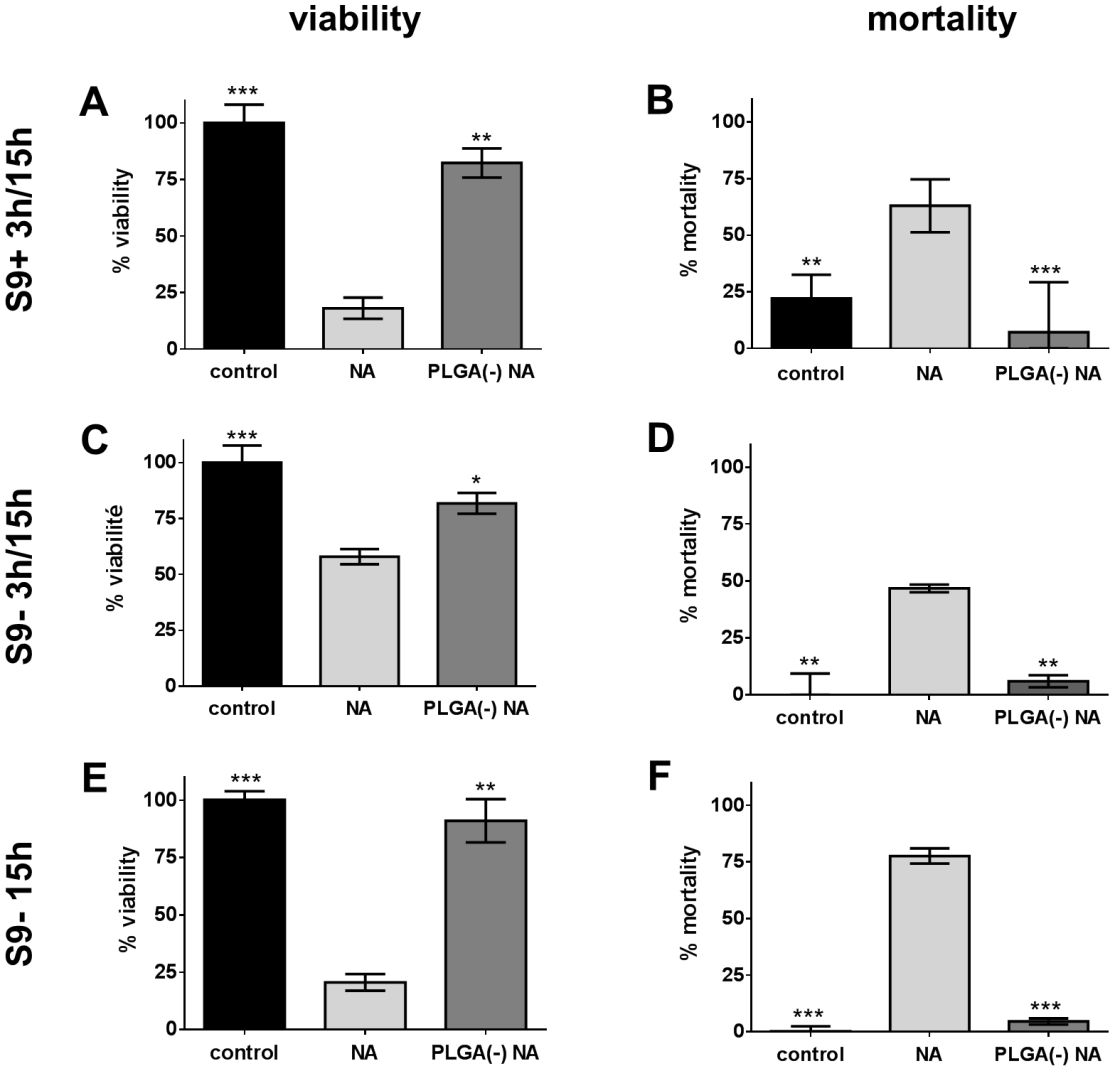
Cytotoxicity of free *versus* PLGA-adsorbed naphthalene. H292 cells were treated with 64.1μg.ml^-1^ (500μM) of free (NA) or nanoparticle-adsorbed naphthalene (PLGA(-) NA) before assessing their viability by MTT assay (A, C, E) and their mortality by LDH assay (B, D) or CellTox Green^®^ assay (F), following three protocols: a 3-hour treatment associated with S9 metabolic activation followed by a 15-hour recovery period (S9+ 3h/15h: A-B), a 3-hour treatment followed by a 15-hour recovery period (S9- 3h/15h: C-D) and a 15-hour treatment (E-F). T-test *: p<0.05; **: p<0.01; ***: p<0.005 *versus* free naphthalene.

The (S9- 3h/15h) exposure schedule with free NA led to a decreased viability (58%) while PLGA(-) NA showed a better viability (82%): thus, only free NA increased cell mortality (47%) (Fig 2C and 2D). The NA activation by S9 mix (S9+ 3h/15h) showed similar but enhanced results: indeed, free NA induced a reduction of viability to 18% and an increase of mortality up to 63%, while PLGA(-) NA results were not affected (viability to 82%) (Fig 2A and 2B). Finally, the (S9- 15h) protocol gave similar results and only free NA was toxic (viability 20%, mortality 77%) (Fig 2E and 2F).

**Fig 3:**
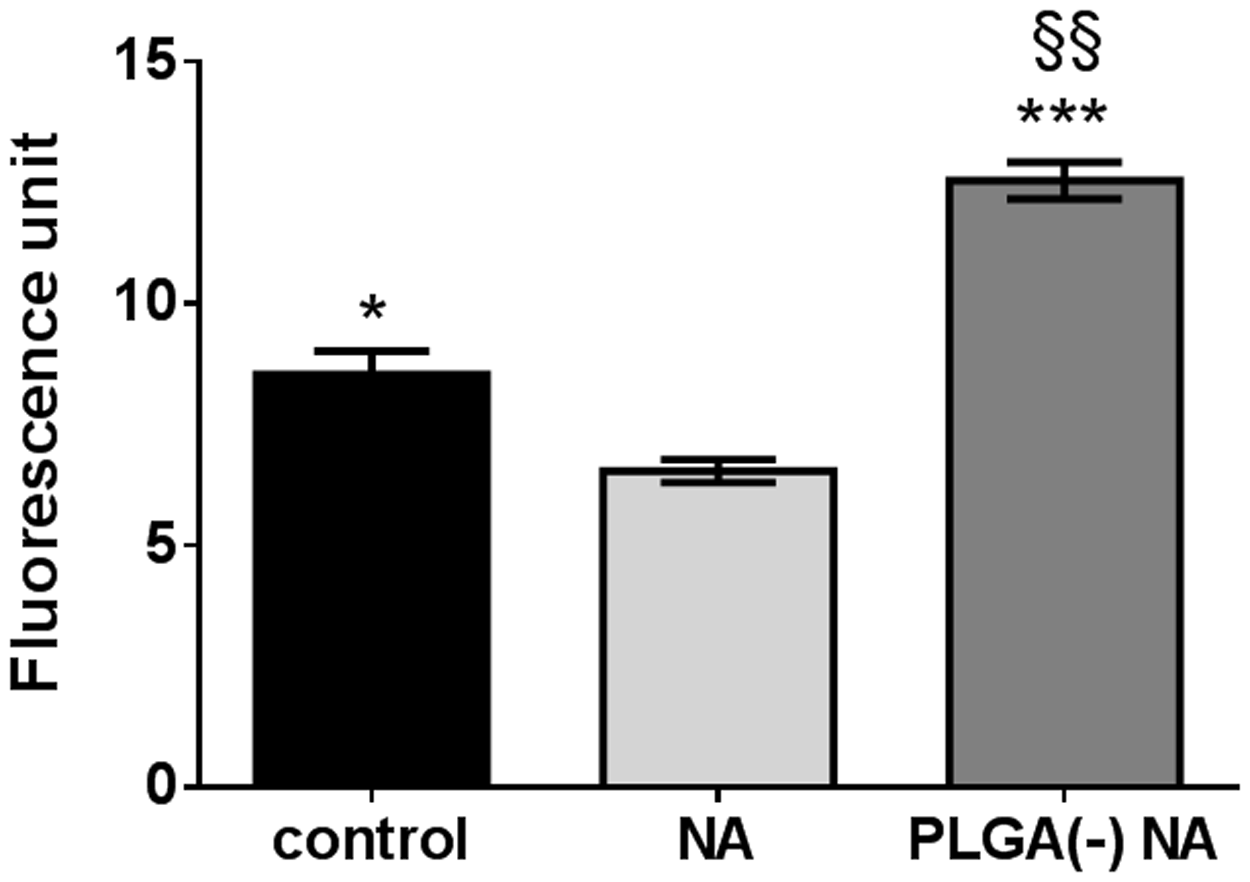
ROS induction by free *versus* PLGA-adsorbed naphthalene. H292 cells were treated for 5 hours with 64.1μg.ml^-1^ (500μM) of free (NA) or nanoparticle-adsorbed naphthalene (PLGA(-) NA) before measuring the ROS induction by the H2DCF-DA method. T-test *: p<0.05; ***: p<0.005 *versus* free naphthalene; §§: p<0.01 *versus* control.

We then examined the influence of free or PLGA-associated NA on reactive oxygen species (ROS) production. We observed that free NA reduced, while PLGA(-) NA increased, the production of ROS (Fig 3). This suggests that this early toxic event is not related to the cytotoxicity observed.

#### Genotoxicity study

The genotoxicity of NA was evaluated by using two complementary approaches: primary DNA damage was measured by standard comet assays and a modified procedure (using hOgg1 endonuclease for the detection of oxidized bases). The micronucleus test was used to assess the induction of chromosomal damage (clastogenicity and/or aneuploidy).

**Fig 4:**
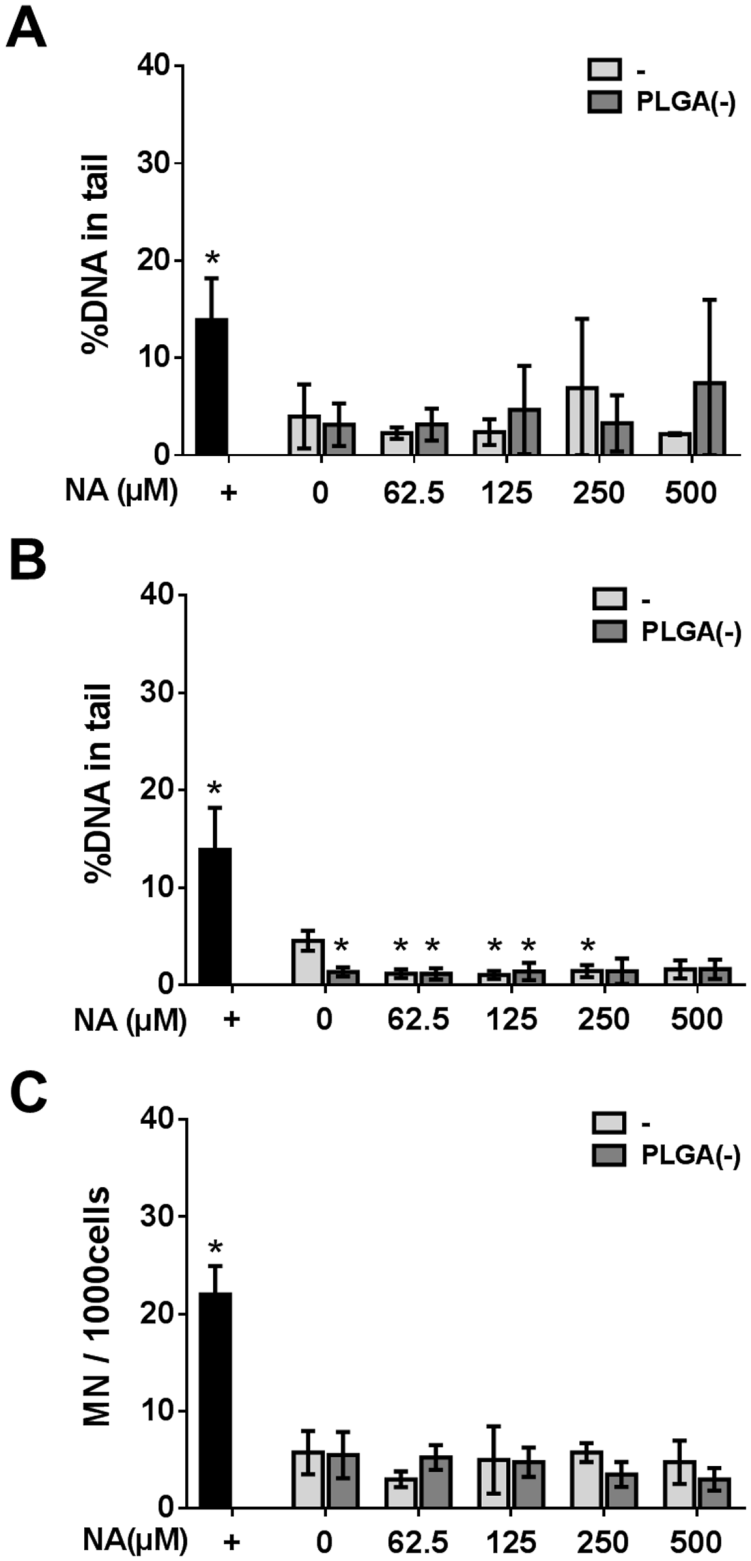
Genotoxicity of free *versus* PLGA-adsorbed naphthalene. H292 cells were treated with 0 to 500μM (64.1μg.ml^-1^) of free (-) or nanoparticle-adsorbed naphthalene (PLGA(-))before assessing the genotoxicity. DNA damage was evaluated by the comet assay (A) and the modified comet assays with the human 8-Oxo-guanine-glycosylase 1 (B). DNA damage is expressed as the percentage of DNA in the tail. Chromosomal aberration was determined by the micronucleus test (C). +: positive control. Mann—Whitney U-test (A, B) *: p<0.05 *versus* the negative control; Chi^2^ test (C) *:p<0.05 *versus* the negative control.

No primary DNA damage was observed while the dose of NA increased from 0 to 500μM and there was no significant difference between free NA and the PLGA(-) NA (Fig 4A). Similar results were obtained with hOGG1 and no increase in DNA damage was observed, confirming that the induction of ROS by PLGA(-) NA was not linked to oxidative DNA damage (Figs 3 and 4B).

The micronucleus test did not show any chromosomal aberrations upon exposure to free or PLGA(-) NA, even at the highest doses, and no difference was observed between free and nanoparticle-adsorbed NA (Fig 4C).

Together, these data showed that nanoparticle-adsorbed NA is not more cytotoxic nor genotoxic than free NA, and that the vectorization of NA by nanoparticles did not enhance the toxicity of this pollutant.

### Vectorization effect on benzo(a)pyrene toxicity

#### Cytotoxicity study and ROS induction

**Fig 5:**
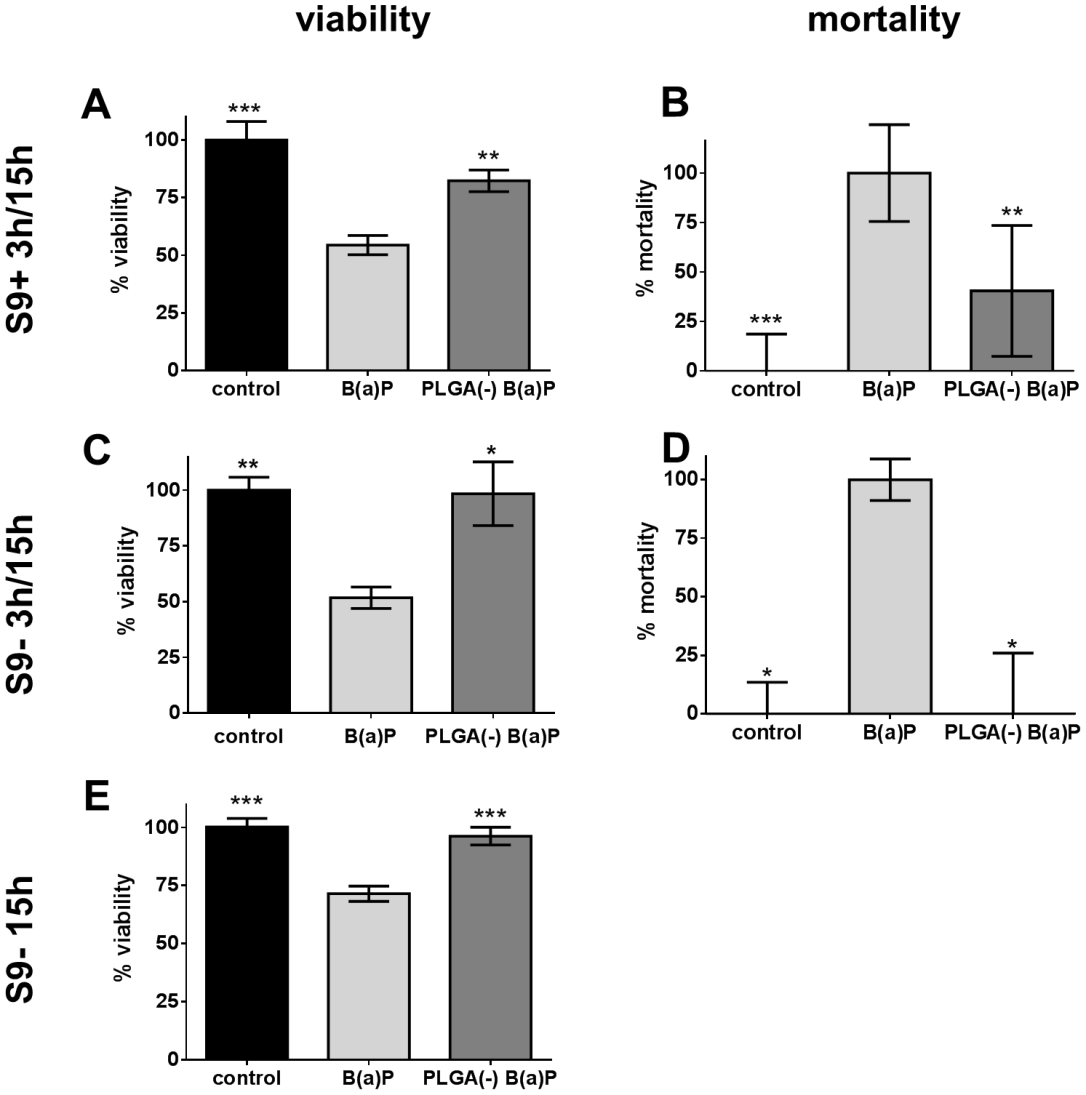
Cytotoxicity of free *versus* PLGA-adsorbed benzo(a)pyrene. H292 cells were treated with 12.6μg.ml^-1^ (50μM) of free (B(a)P) or nanoparticle-adsorbed benzo(a)pyrene (PLGA(-) B(a)P) before assessing the viability by MTT assay (A, C, E) and the mortality by LDH assay (B, D) following three protocols: a 3-hour treatment associated with S9 metabolic activation followed by a 15-hour recovery period (S9+ 3h/15h: A-B), a 3-hour treatment followed by a 15-hour recovery period (S9- 3h/15h: C-D) and a 15-hour treatment (E). T-test *: p<0.05; **: p<0.01; ***: p<0.005 *versus* free benzo(a)pyrene.

The cytotoxicity of free *versus* nanoparticle-adsorbed B(a)P (PLGA(-) B(a)P)) was studied. The (S9+ 3h/15h) condition showed that B(a)P decreased the cell viability to 54.46% while PLGA(-) B(a)P only reduced the cell viability to 82.32%. The LDH assay gave consistent results with 100% mortality for free B(a)P and only 40.43% for PLGA(-) B(a)P (Fig 5A and 5B). Similar results were obtained without metabolic activation (S9- 3h/15h) (Fig 5C and 5D). Lastly, with the (S9- 15h) condition, a moderate cytotoxicity was observed only for free B(a)P (71.49% of viability) compared with PLGA(-) B(a)P (Fig 5E).

**Fig 6:**
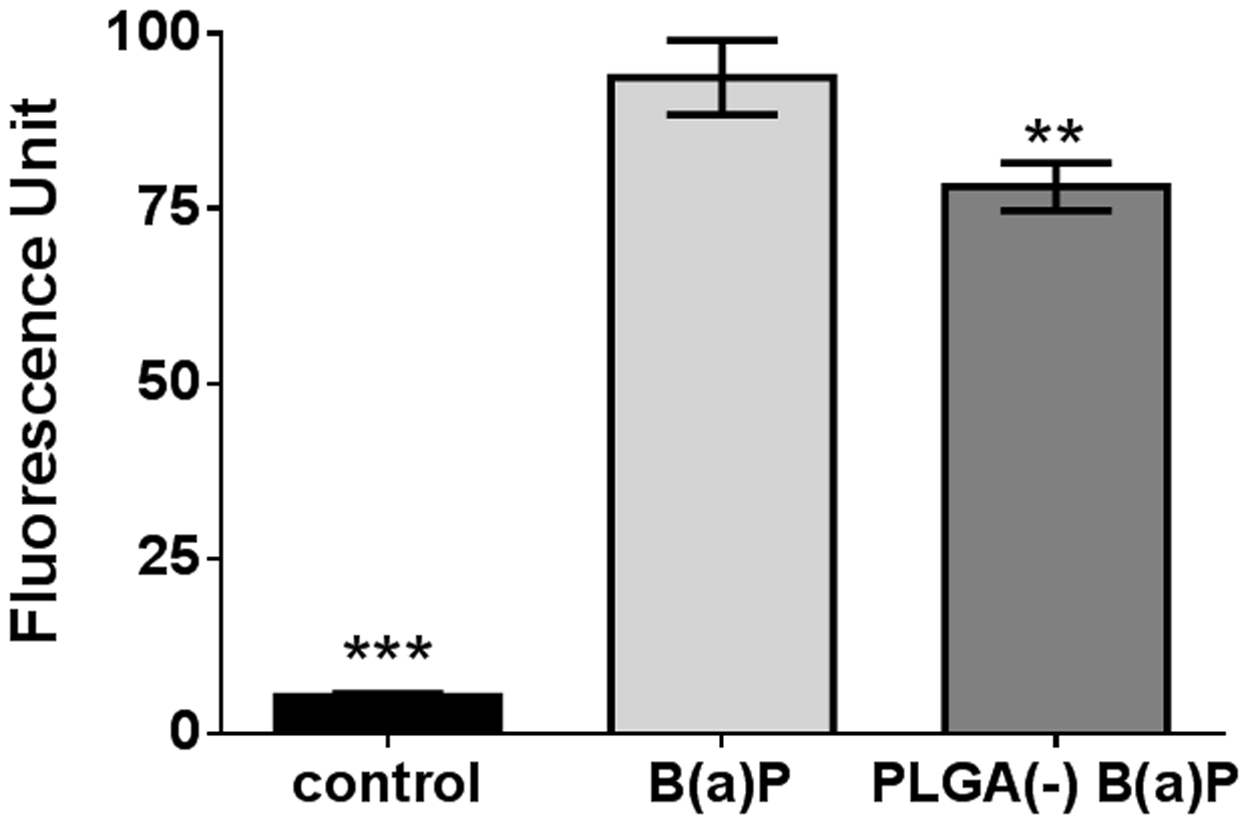
ROS induction by free *versus* PLGA-adsorbed benzo(a)pyrene. H292 cells were treated for 5 hours with 12.6μg.ml^-1^ (50μM) of free (B(a)P) or nanoparticle-adsorbed benzo(a)pyrene (PLGA(-) B(a)P) before measuring the ROS induction by the H2DCF-DA method. T-test **: p<0.01; ***: p<0.005 *versus* free benzo(a)pyrene.

ROS production was then studied and after 5 hours of treatment without metabolic activation, high inductions were observed for both free B(a)P (17.5 fold over control) and PLGA(-) B(a)P (14.5 fold over control). The difference in ROS induction between free and nanoparticle-adsorbed B(a)P was significant, suggesting that nanoparticle-adsorbed B(a)P is less toxic than free B(a)P (Fig 6).

#### Genotoxicity study

**Fig 7:**
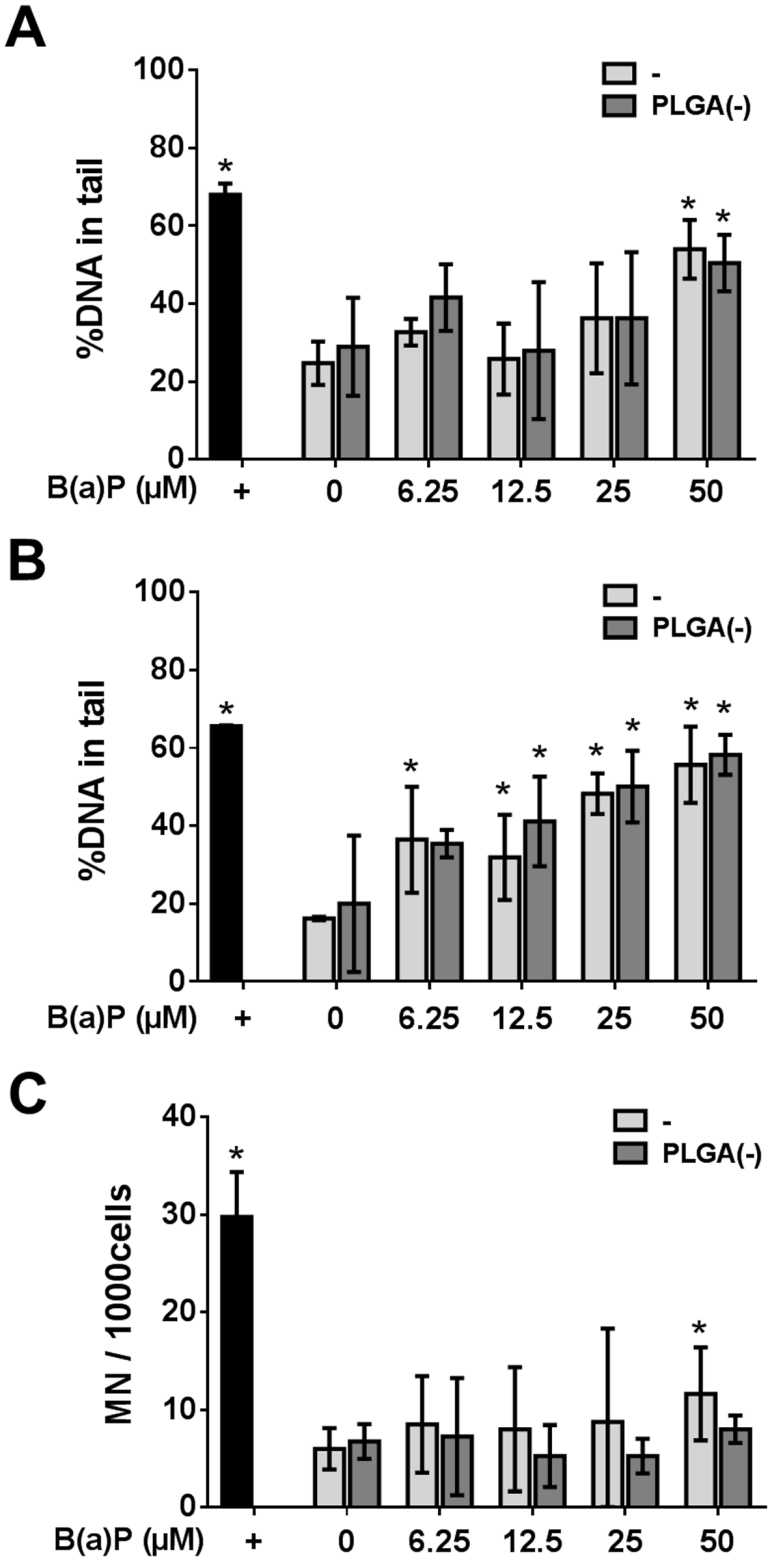
Genotoxicity of free *versus* PLGA-adsorbed benzo(a)pyrene. H292 cells were treated with 0 to 50μM (12.6μg.ml^-1^) of free (-) or nanoparticle-adsorbed benzo(a)pyrene (PLGA(-))before assessing the genotoxicity. DNA damage was evaluated by the comet assay (A) and the modified comet assays with the human 8-Oxo-guanine-glycosylase 1 (B). DNA damage is expressed as the percentage of DNA in the tail. Chromosomal aberration was determined by the micronucleus test (C). +: positive control. Mann—Whitney U-test (A, B) *: p<0.05 *versus* the negative control; Chi^2^ test (C) *:p<0.05 *versus* the negative control.

The comet assay revealed a progressive increase in primary DNA damage with dose of PLGA(-) B(a)P, significant at the highest dose (50μM) (Fig 7A). The modified comet assay demonstrated a progressive increase in oxidative-dependent DNA damage, but was not related to the adsorption of B(a)P onto PLGA nanoparticles. It is noteworthy that at the dose of 6.25μM, oxidative-dependent primary DNA damage was induced by the free B(a)P but not by the nanoparticle-associated B(a)P (Fig 7B). Finally, at the highest dose (50μM), free B(a)P gave rise to chromosomal aberrations while nanoparticle-associated B(a)P did not (Fig 7C).

These results showed that free B(a)P is more cytotoxic and genotoxic than nanoparticle-adsorbed B(a)P, and that the vectorization of B(a)P by nanoparticles did not enhance the toxicity of this pollutant.

### Vectorization effect on di-ethyl-hexyl-phthalate toxicity

#### Cytotoxicity study and ROS induction

**Fig 8:**
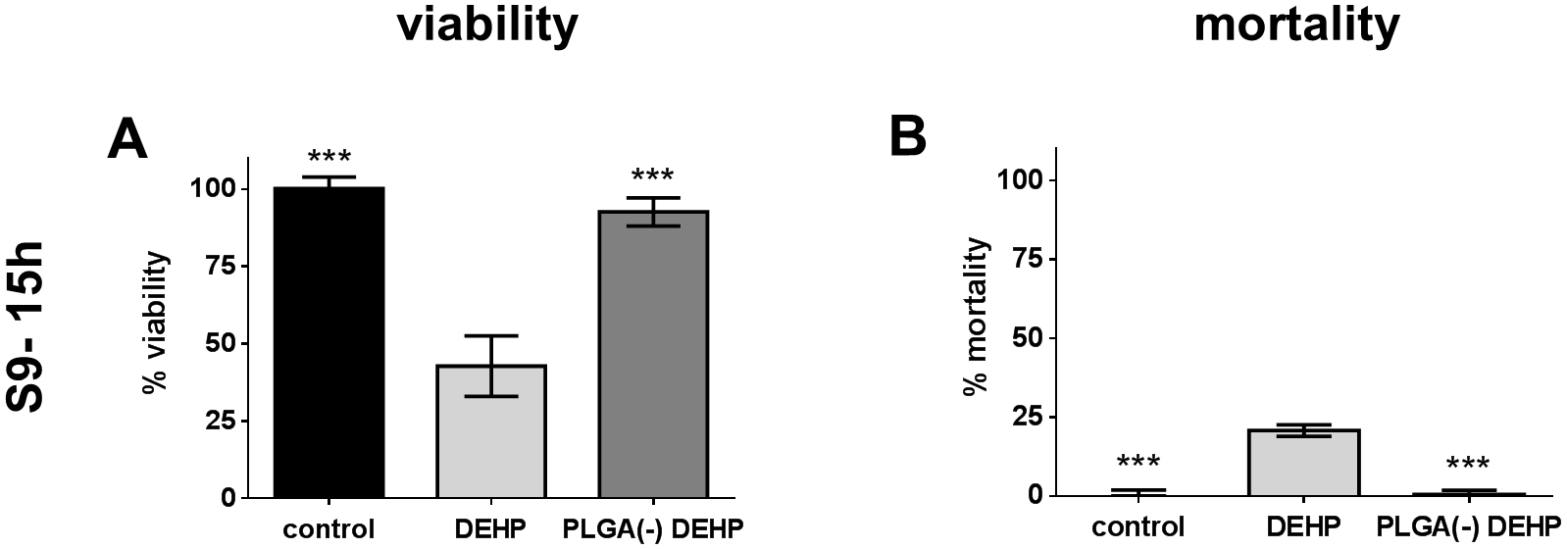
Cytotoxicity of free *versus* PLGA-adsorbed di-ethyl-hexyl phthalate (DEHP). H292 cells were treated for 15 hours with 58.6μg.ml^-1^ (150μM) of free (B(a)P) or nanoparticle-adsorbed benzo(a)pyrene (PLGA(-) B(a)P) before assessing the viability by MTT assay (A) and the mortality by CellTox Green^®^ assay (B). T-test ***: p<0.005 *versus* free DEHP.

**Fig 9:**
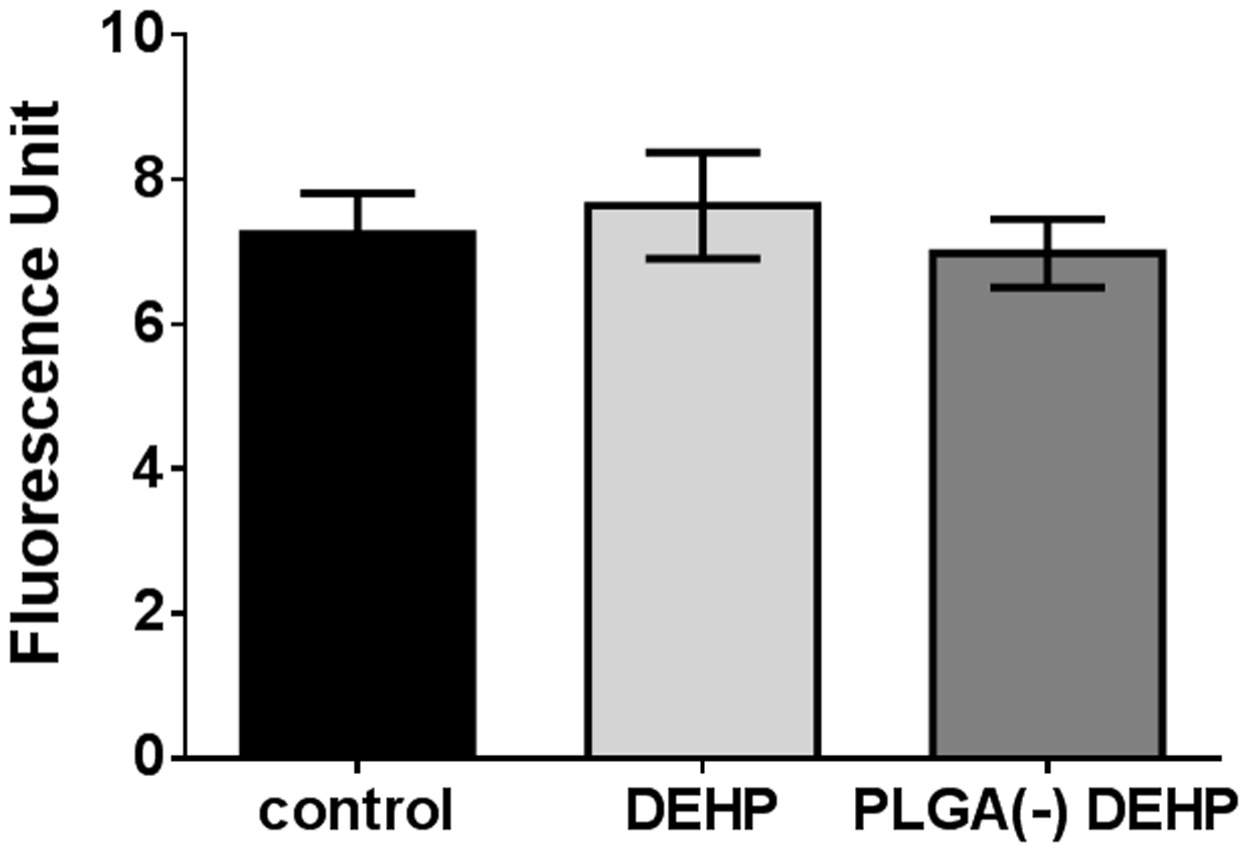
ROS induction by free *versus* PLGA-adsorbed di-ethyl-hexyl phthalate (DEHP). H292 cells were treated for 5 hours with 58.6μg.ml^-1^ (150μM) of free (DEHP) or nanoparticle-adsorbed pollutant (PLGA(-) DEHP) before measuring the ROS induction by the H2DCF-DA method. T-test no significant *versus* free DEHP.

Since DEHP is not sensitive to metabolic activation by the S9 mix, only the (S9- 15h) condition was studied. Free DEHP induced a decrease in viability (42.77%) and an increase in mortality (20.77%), while nanoparticle-associated DEHP (PLGA(-)DEHP) did not exhibit an effect on either parameter (92,65% viability and no increase in mortality) (Fig 8A and 8B). This was not related to oxidative damage since no ROS production was induced (Fig 9). Thus, the cytotoxicity of DEHP was absent when it was associated with nanoparticles.

#### Genotoxicity study

**Fig 10:**
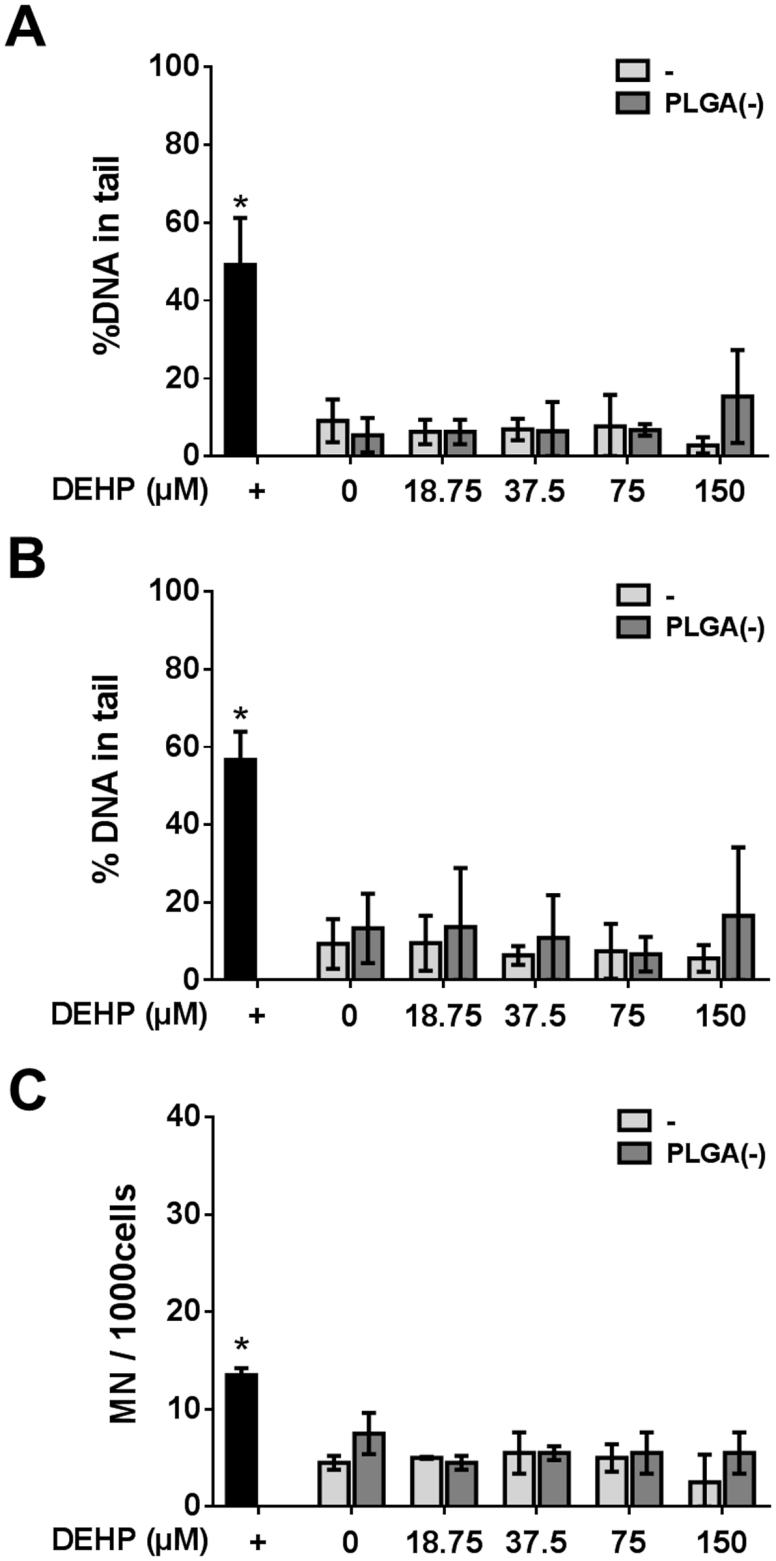
Genotoxicity of free *versus* PLGA-adsorbed di-ethyl-hexyl phthalate (DEHP). H292 cells were treated with 0 to 150μM (58.6μg.ml^-1^) of free (-) or nanoparticle-adsorbed pollutant (PLGA(-) DEHP) before assessing the genotoxicity. DNA damage was evaluated by the comet assay (A) and the modified comet assays with the human 8-Oxo-guanine-glycosylase 1 (B). DNA damage is expressed as the percentage of DNA in the tail. Chromosomal aberration was determined by the micronucleus test (C). +: positive control. Mann—Whitney U-test (A, B) *: p<0.05 *versus* the negative control; Chi^2^ test (C) *:p<0.05 *versus* the negative control.

The genotoxicity of PLGA(-)DEHP was assessed by the two methods described above. No primary DNA damage was observed for any of the doses tested (Fig 10A and 10B). Moreover, neither free nor nanoparticle-adsorbed DEHP induced chromosomal aberrations (Fig 10C).

These results demonstrate that free DEHP is cytotoxic, contrary to the nanoparticle-adsorbed form, and that the vectorization of DEHP by nanoparticles reduced the toxicity of this pollutant.

## Discussion

In an effort to understand the role that nanoparticle-dependent vectorization could play in the toxicity of airborne atmospheric pollutants, we analyzed the cyto- and geno-toxicity of three common airborne pollutants (B(a)P, NA and DEHP), either in their free state or adsorbed onto PLGA(-) nanoparticles.

The choice of the nanoparticle model used here was based on its safety, on the absence of induced toxicity at high dose, and on a production method free of chemical residues so as to limit the potential toxic side-effects of compounds and solvents added during the synthesis. We previously developed a single-step production of PLGA nanoparticles [35], without the use of detergent, and that does not exhibit cyto- or genotoxicity [30]. Moreover, the PLGA copolymer is FDA approved. Another aspect of the selection was the possibility to adsorb the selected pollutants, favored by the hydrophobicity of the copolymer. All these criteria made PLGA(-) an interesting nanoparticle model for adsorption of the selected pollutants considered in this work.

The selected cell line mimics an airway epithelium and possesses the catabolic enzymes able to metabolize the pollutants, such as the P450 cytochrome family. The 16HBE14o- cell line was not sensitive to B(a)P-induced cell death and was consequently rejected. The A549 cell line, a lung alveolar-based carcinoma cell line, was less sensitive to B(a)P than the NCI-H292 cell line, derived from a mucoepidermoid pulmonary carcinoma. In addition, the expression of enzymes involved in xenobiotic catabolism has also been described for this latter cell line [44, 45], therefore we retained the NCI-H292 cell line for this study. This cell line is a bronchial epithelial model and it is described that nanoparticles and UFP preferentially target the alveolar part of the lung. However, considering the reduced available surface of the conducting airways, the deposition efficiency of the smallest particles remains high and a bronchial epithelial model thus remains pertinent [9].

In our study on the impact of the vectorization by nanoparticles of volatile pollutants on their toxicity, a submerged model was developed. This model is consistent with the use of PLGA(-) nanoparticles that are synthesized and characterized in water. This also allowed nanoparticles to reach the cells to study the vectorization of the selected pollutants. Indeed, the deposition mechanism of nanoparticles is limited in these *in vitro* conditions but favorably balanced by the Brownian motion and the ionic and hydrophobic interactions with the cells. Moreover, in this cell culture model, the PLGA(-) nanoparticles do not aggregate but retain their size and zeta potential [35], meaning that vectorization remains optimal throughout the analyses.

We studied three different pollutants and associated them by a simple mixing with the nanoparticles, leading to the adsorption of these pollutants onto the nanoparticles’ surface. This allowed the pollutants to be transported by the nanoparticles and to access the cellular environment, and through avoidance of chemical means to achieve these formulations, thereby limit any toxic side-effects. Size and zeta potential measurements exhibited strong association between pollutants and nanoparticles, and this was confirmed by HPLC analyses showing about 100% association for NA and B(a)P, and 85% association for DEHP (Tables 1 and 2). This meant that a fraction of DEHP (15%) was nevertheless in a free state in the PLGA(-) DEHP formulation. This fraction had no measurable effect, however, since there was no difference between controls and PLGA(-) DEHP treated cells, unlike for free DEHP (Figs 8 and 9).

The three pollutants, either in a free state or adsorbed onto nanoparticles, showed specific behavior when viability and ROS induction were compared. When associated to PLGA(-), all pollutants exhibited less cytotoxicity than when cell viability was measured after exposure to the free state pollutants. While free NA induced less ROS than PLGA(-) NA (Fig 3), the opposite was observed for B(a)P (Fig 6), and no ROS was induced by free DEHP (Fig 9). These results indicate a lack of a consistent relationship between ROS induction and cytotoxicity.

Concerning genotoxicity, the three pollutants also exhibited specific behavior. No oxidative-dependent DNA damage was observed for either free or PLGA-associated NA, while an increase in ROS induction was observed for PLGA(-) NA (Figs 3 and 4). The B(a)P treatment led to DNA damage relative to the dose (not significant), but with no effect of the association of B(a)P to PLGA(-). However, regarding chromosomal aberrations, adsorption onto PLGA(-) decreased the toxicity of B(a)P for the highest dose (Fig 7). Finally, DEHP did not induce genotoxicity (Fig 10). Thus, despite the different behavior of the three selected pollutants, our results show that adsorption of pollutants onto inert nanoparticles does not lead to an increased toxicity, even if ROS induction was increased in the case of NA. Since the developed nanoparticles did not aggregate in biological fluids [35], these differences could not be related to a specific deposition mechanism.

Taken together, these results generate the hypothesis that the toxicity in airborne atmospheric pollution is not due to the vectorization of adsorbed pollutants by UFP. They also suggest that previous studies on the toxicity of the isolated particulate fractions attributed toxicity to particulates as a result of the intrinsic toxicity of the particles studied [14, 17].

The cellular availability of nanoparticle-associated airborne pollutants could be limited compared to volatile pollutants. Indeed, despite the fact that nanoparticles may reach deep into the lung, the pollutant in a free, unbound, state may be expected to have better access to all the compartments of the airway epithelium, including the deepest part of the lung. On the other hand, it is well known that PLGA nanoparticles enter cells by endocytosis [46]. Thus, the association of pollutants to nanoparticles could limit their bio-availability, and/or their intracellular toxicity could be limited by the endocytosis of the nanoparticle-associated pollutant into an unexpected compartment. In this way, the pollutant is less accessible to the catabolic enzymes of xenobiotics that may generate their toxic metabolites, or alternatively, the degradation of the pollutant is favored, thereby limiting its toxicity.

In this regard, UFP (or nanoparticles) with no intrinsic toxicity and acting only as a vector of gaseous pollutants could protect cells in the deep lung from pollutants’ toxicity. However, further studies are needed to completely validate this hypothesis and to transpose it to natural atmospheric pollution.

To conclude, our results support observations that the toxicity in airborne atmospheric pollution is not directly related to the adsorption of volatile pollutant on particles, since their vectorization does not exacerbate the toxicity of adsorbed pollutants. The vast diversity of airborne contaminants has introduced significant challenges for risk assessment and management. Strategies have been developed to evaluate and manage unintentional human exposure, particularly occupational exposure. Although this requires further investigation, our work shows possible implementation of risk management of inhaled pollutants. In order to better manage the risk associated to pollution on health, attention should be paid to the analysis of complex pollutant mixtures, and should take into account, among other things, the ability of particles to transport adsorbed pollutants into cells.

## Conclusion

Atmospheric pollution is a complex mixture of volatile pollutants and particulate matter. Airborne particles can adsorb gaseous pollutants and these complexes are usually studied together. Deciphering the toxic effect of each component in these complexes, in addition to mixtures of vapor phase pollutants and the particles themselves, as well as particle-associated pollutants, remains extremely challenging. During ultrafine particle pollution episodes, authorities alert the general population about potential health concerns, but tend not to explain that the particles are not the only reason for any increase in potential toxicity: indeed, particulate pollution peaks are often coupled with an exceedance of regulatory limits for several pollutants in both vapor and particulate phases. Here, we have suggested that the vectorization of vapor phase pollutants by fine particles may limit the toxicity of the pollutants and thus may eventually be considered protective, and that the evaluation of the toxic effect of atmospheric pollution needs to be studied for each individual compound and the particles to which they are adsorbed, as well as for pollutant-particle complexes.
